# Embryologic Association of Tornwaldt's Cyst with Cerebral Artery Abnormalities and Infarction: A Case Report

**DOI:** 10.1155/2012/129503

**Published:** 2012-10-08

**Authors:** Michael F. Osborn, Benjamin K. Buchanan, Nassim Akle, Ahmed Badr, Jun Zhang

**Affiliations:** ^1^COE in Neurosciences and Departments of Anesthesiology and Biomedical Sciences, Texas Tech University Health Science Center, El Paso, TX 79905, USA; ^2^Department of Radiology, Texas Tech University Health Science Center, El Paso, TX 79905, USA

## Abstract

*Background and Purpose*. Tornwaldt's cysts are rare nasopharyngeal lesions that develop from remnants of the embryonic notochord. *Summary of Case*. We reported a twelve-year-old female stroke patient with Tornwaldt's cysts, whose father also suffered a stroke at age fifty two with the presence of an abdominal aortic aneurysm, suggesting a genetic influence in this case. *Conclusions*. This paper suggests an etiologic connection between Tornwaldt's cysts and cerebral vasculature abnormalities by way of notochordal dysfunction during development, likely the result of perturbation of notochord-derived molecular cues during development or biogenesis.

## 1. Introduction

Tornwaldt's cysts, also known as pharyngeal bursae, are rare midline cysts found in the nasopharynx [1]. They are formed as a remnant of the embryonic notochord at its interface with the embryonic foregut [1]. They are found cranial to the superior constrictor muscles and are covered by the mucous membrane of the nasopharynx [2]. These cysts are usually incidental findings on imaging of the head and neck region [2]. These cysts are generally diagnosed during imaging investigations for symptoms of sore throat, headache, dizziness, seizure, foul oral odor or taste, purulent discharge, or eustachian tube obstruction, all of which may occur due to infection of these cysts [3]. The reported incidence of Tornwaldt's cysts range from .06% to as high as 5% of autopsy cases or imaging films, with peak incidence in the second or third decade of life [4].

 The notochord plays an important role in the development of the embryo. The embryologic origin of Tornwaldt's cysts derives from the period of transformation of the notochordal process into the solid notochord between days 16 and 22 of development [1]. During this time, the ventral wall of the notochordal process fuses with the endoderm to form a notochordal plate [5]. This process begins at the caudal end and progresses cranially [5]. After the plate is formed at the cranial end, the notochordal plate infolds and forms a solid rod, the notochord [6]. Failure of complete infolding leads to the development of a Tornwaldt's cyst, a remnant of the notochord attached to the endoderm.

The notochord is located at the center of the developing embryo, parallel with its elongated plane, forming the central axis of the developing embryo [7]. As the central axis, the notochord's role is to induce and pattern the development of the early embryo [8]. This occurs through various cellular signaling mechanisms, including hedgehog (Hh) and chordin [8]. The notochord is also involved in dorsal-ventral patterning and induction of the development of various tissues [8].

**Figure 1:**
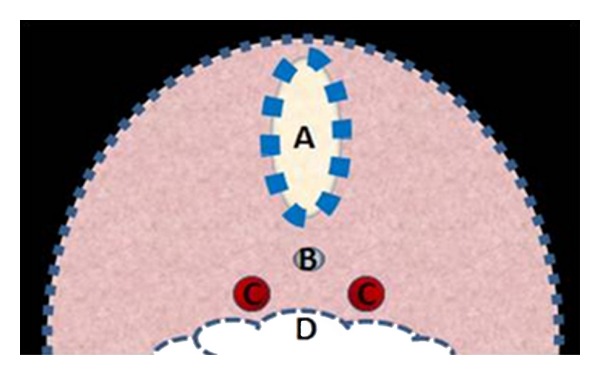
Depiction of a cross section through the early embryo demonstrating the relationship between the neural tube (A), notochord (B), dorsal aortae (C), and gut tube (D).

In addition to the proximity to both the gut and neural tube, the notochord is in close proximity to the developing vasculature, most notably the dorsal aortae. The dorsal aortae are two large vessels that run the length of the embryo [9]. These vessels lie on either side and slightly ventral to the notochord, and dorsal to the gut tube (Figure 1) [9]. The cranial extensions of the dorsal aortae form the distal portions of the internal carotid artery including the middle cerebral arteries [10]. In addition to these cranial vessels, the dorsal aortae fuse to form the descending aorta [10].

## 2. Patients and Methods

A twelve-year-old Hispanic female, born in the United States, presented to the hospital for resection of a brain tumor. The patient had previously sought treatment in Mexico for complaints of facial drooping, drooling, and seizures, where she was worked up for seizure disorder and treated with valproic acid. The patient's magnetic resonance imaging (MRI) showed a lesion, suspecting to be a brain tumor. The patient was referred to our clinic for surgical removal of the suspected tumor.

**Figure 2:**
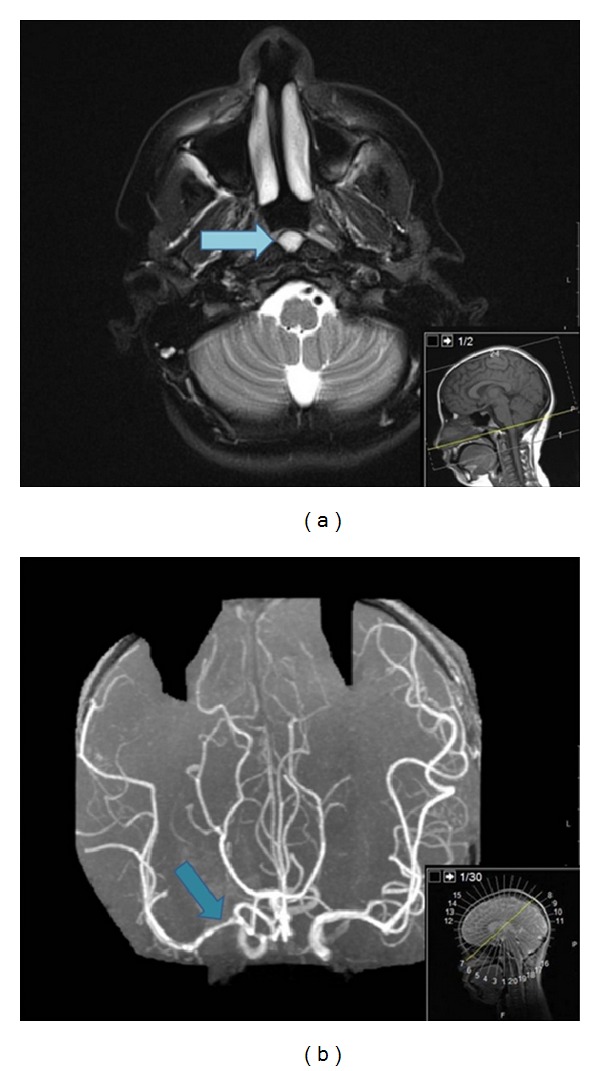
Magnetic resonance images from patient depicting abnormal findings. (a) Axial T2 weighted MR image through the nasopharynx demonstrating a small well-circumscribed T2 hyperintense, CSF-like cyst nestled between the longus colli muscles deep to the nasopharyngeal mucosa consistent with an uncomplicated Tornwaldt's cyst (arrow). (b) 3D time-of-flight MRA of the intracranial circulation demonstrating narrowing and irregularities with the wall of the M1 segment of the right middle cerebral artery (arrow) indicating, but not limited to, arteritis or dissection.

Upon admission, the patient again complained of facial drooping and drooling in addition to left-sided upper extremity weakness, bilateral ear pain, right-sided facial throbbing, a pulsating headache, and episodic dizziness. The patient had experienced similar episodes for the past few months in addition to seizures for several years. The patient's father reported suffering a stroke at age fifty-two and the presence of an abdominal aortic aneurysm. The remainder of the family history was noncontributory. Upon physical examination, the patient was found to have pronator drift, 2+ deep tendon reflexes, boggy nasal turbinates, and intact cranial nerves two through nine. No systemic symptoms were noted. Upper extremity blood pressure was measured initially at 97/42 and later at 105/64 (50th percentile for a 12 year old is 106/52 [11]). An EEG was normal. Blood tests showed anti-nuclear antibody negative. Further preoperative MRI demonstrated displacement of the right lateral ventricle from mass effect, a focal area of abnormal signal intensity extending from the ventricle to the centrum semiovale and internal carotid artery, and enhancement in the caudate and putamen. These findings suggested an infarct rather than a tumor. A follow-up MRI without contrast demonstrated remodeling of the stroke area and a Tornwaldt's cyst was also noted (Figure 2). A 3D time-of-flight magnetic resonance angiography was then performed which showed narrowing and irregularity of the M1 segment of the right middle cerebral artery (Figure 2). From these images, the patient was diagnosed with arteritis, Bell's palsy, and Tornwaldt's cyst. The patient was discharged from the hospital with a treatment plan for arteritis.

## 3. Discussion

The discharge diagnosis of this patient was officially listed as cerebral infarction due to arteritis. As evidenced from the case presentation, there was very little information to conclusively demonstrate the diagnosis of arteritis. Because the middle cerebral artery was involved in this case, we suspect either temporal or Takayasu's arteritis (both types of large-vessel arteritis) as the proposed diagnosis. Clinical suspicion of arteritis begins as a result of various constitutional symptoms due to systemic inflammation. These nonspecific symptoms include fever, malaise, memory impairment, anorexia, weight loss, fatigue, and depression [12]. The definitive classification for temporal arteritis is based on 1990 American College of Rheumatology criteria which states that the patient must have three of the following five items present: age of onset older than fifty years, new onset headache or localized head pain, temporal artery tenderness to palpation or reduced pulsation, erythrocyte sedimentation rate (ESR) greater than 50 mm/h, and/or abnormal arterial biopsy [13]. The diagnosis for Takayasu's arteritis is based on the European League Against Rheumatism criteria which states that a patient must have angiographic abnormalities in addition to decreased peripheral artery pulses, claudication of extremities, blood pressure difference in the four limbs of more than 10 mm Hg, bruits over the aorta or its major branches, or hypertension [14]. Of these diagnostic criteria, the only evidence suggesting arteritis in this patient was the angiographic abnormality seen on MRI and the localized head pain. The patient did not meet the criteria needed to make the diagnosis, lacked systemic symptoms generally described in patients with arteritis, and only had a focal vessel abnormality. 

Based on the embryology of the notochord and its relationship to the forming vasculature during early development, we propose a link between this patient's Tornwaldt's cyst and the irregularity in the M1 segment of the middle cerebral artery (MCA). As discussed previously, the notochord plays an essential role in establishing the center of the embryo through various signaling mechanisms. Notochord-derived hedgehog (Hh) is a key molecular cue for the specification and differentiation of multiple tissues and organs during embryonic development. For vasculogenesis/angiogenesis, Hh also plays an important role for appropriate blood island formation in yolk sac vessel development [15], endothelial tube formation [16], and yolk sac angiogenesis in the murine embryo [17] and modulation of angioblastic migration from the posterior lateral mesoderm [18], initiation of artery-specific gene expression [19, 20], and the earliest stages of endocardial specification in zebrafish [21].Perturbation of the notochord, similar to the events causing the formation of a Tornwaldt's cyst during development, could result in dysfunction of endoderm development or of cell signaling via Hh during vasculogenesis/angiogenesis. Through the remnant of the notochord in the gut tube, there could be further interaction with the dorsal aortae at the level of the remnant through persistent, inappropriate, or absent cell signaling in this region. These defective processes could lead to a cystic or atretic deformity in the MCA. Similar deformities have been demonstrated in chicken and quail embryos, in addition to delayed fusion of the dorsal aortae and impaired assembly of endothelial cells into vascular tubes in the dorsal aortae after inhibition of Hh signaling [10]. This defective signaling would lead to abnormal vasculogenesis/angiogenesis, which would predispose an individual to stroke. This hypothesis is further supported in this case by the family history. The patient's father had a stroke at fifty-two years of age and a large abdominal aortic aneurysm (possible abnormal vasculogenesis during early development). The relationship of the descending aorta to the notochord is similar to that of the MCA, as previously described. It is possible that this patient's father had a similar condition leading to deformation of vessels in both the descending aorta and the cerebral vasculature. In sum, this provides evidence for the association between notochordal dysfunction (as in Tornwaldt's cyst) and vascular abnormalities.

## 4. Conclusion

This case presentation suggests a possible etiologic connection between Tornwaldt's cyst and cerebral vasculature abnormalities by way of notochordal signaling perturbation, a predisposition to cerebral vascular accidents. This association between Tornwaldt's cysts and stroke has not been discussed in the literature, as demonstrated by an online literature search using PubMed (“Tornwaldt's” and “Epidemiology” or “Risk Factor” or “Association” or “Stroke”) which revealed no published reports to date that match the criteria. Based on this case presentation, we postulate that the patient had a genetic defect in the notochord-derived hedgehog signaling pathway related to vascular development.
